# Managing Acute Agitation at the Edge of Frailty: A Global Systematic Review of Rapid Tranquillisation in Older Psychiatric Inpatients

**DOI:** 10.1192/bjo.2026.11132

**Published:** 2026-06-30

**Authors:** Sathyan Soundara Rajan, Sneh Babhulkar, Gaurav Uppal, Linda Thomas, Asha Dhandapani

**Affiliations:** 1 BCUHB, Wrexham, United Kingdom; 2 NHS Greater Glasgow and Clyde, Glasgow, United Kingdom; 3 Satyam Hospital, Ludhiana, India; 4 Sheffield Health Partnership University NHS Trust, Sheffield, United Kingdom

## Abstract

**Aims:**

Rapid tranquillisation (RT) is widely used to manage acute agitation in psychiatric inpatient settings when non-pharmacological strategies fail. Older adults represent a particularly vulnerable population due to frailty, multimorbidity, polypharmacy, and age-related pharmacokinetic and pharmacodynamic changes, which increase the risk of adverse drug events.

Despite routine clinical use, most evidence regarding RT practice is derived from mixed-age adult populations, with limited age-specific guidance for elderly psychiatric inpatients. This systematic review aims to evaluate the evidence available and assess its applicability to older adults.

Aim was to systematically review and evaluate global evidence on the efficacy and safety of pharmacological rapid tranquillisation strategies in older psychiatric inpatient settings.

**Objectives:**

1. To compare the effectiveness of different RT pharmacological agents and combinations.

2. To evaluate reported adverse outcomes associated with different RT pharmacological agents.

3. To identify gaps in age-specific evidence, targeting older persons and international practice variations.

**Methods:**

A systematic literature search of PubMed, EMBASE and related databases, Cochrane Library, grey literature and reference lists was conducted following PRISMA 2020 guidelines. Randomised control trials evaluating pharmacological RT agents for acute agitation in psychiatric or emergency settings were included.

Outcomes of interest included sedation efficacy, time to tranquillisation and adverse events. Risk of bias was assessed using the Cochrane RoB 2 tool. Due to inconsistent reporting of quantitative outcomes and a lack of elderly-specific data, a formal meta-analysis was not done.

**Results:**

Fifteen high-quality RCTs were identified, all involving mixed-age adult populations, none exclusively enrolled older psychiatric inpatients (>65 years).

Midazolam consistently demonstrated a faster onset of sedation compared with antipsychotic or benzodiazepine monotherapy. Combination regimens, particularly haloperidol plus promethazine or lorazepam, were more effective than monotherapy in achieving rapid tranquillisation and reducing the need for additional medication. Adverse events were generally infrequent; however, haloperidol monotherapy was associated with a higher rate of extrapyramidal symptoms, and midazolam with transient respiratory depression in some studies.

**Conclusion:**

The absence of RCTs that are age-specific, targeting older persons, limits the direct applicability of existing evidence to older psychiatric inpatients. Current RT practicesfor older adults are largely extrapolated from general adult data, despite the heightened safety risks associated with this population. Inconsistent outcome reporting further restricts quantitative analysis.

There is a critical lack of elderly-specific evidence to guide rapid tranquillisation in psychiatric inpatient settings. Future research should prioritise age-stratified trials with standardised outcome reporting to inform safer, evidence-based practice.

